# Nursing students’ perceptions of nonverbal communication using chronemics in surgical settings

**DOI:** 10.1590/0034-7167-2025-0359

**Published:** 2026-09-28

**Authors:** Albert Lengruber de Azevedo, Sílvia Teresa Carvalho de Araujo, Paulo Sergio da Silva, Francisco Gleidson de Azevedo Gonçalves

**Affiliations:** I Universidade Federal de Roraima. Boa Vista, Roraima, Brazil; II Universidade Federal do Rio de Janeiro. Rio de Janeiro, Rio de Janeiro, Brazil; III Universidade Federal de Lavras. Lavras, Minas Gerais, Brazil; IV Instituto Estadual de Hematologia Arthur de Siqueira Cavalcanti. Rio de Janeiro, Rio de Janeiro, Brazil

**Keywords:** Perception, Students, Nursing, Nonverbal Communication, Surgical Centers, Hospital, Education, Nursing., Percepción, Estudiantes de Enfermería, Comunicación No Verbal, Centro Quirúrgico Hospitalario, Educación en Enfermería.

## Abstract

**Objectives:**

to understand nursing students’ perceptions of nonverbal communication using chronemics in surgical settings.

**Methods:**

a qualitative, descriptive-exploratory study, based on Edward T. Hall’s theory of nonverbal communication. Thirty nursing students from a public institution in northern Brazil participated. Data were collected in May and June 2025 through semi-structured interviews and analyzed using thematic content analysis.

**Results:**

chronemics was perceived as a central element in surgical settings. The accelerated pace generated strangeness, anxiety, and insecurity, hindering learning. The waiting period revealed the team’s priorities, indicating who receives attention in the care flow. The nursing staff’s time management highlighted the conflict between task efficiency and availability for teaching, establishing itself as a training model.

**Final Considerations:**

chronemics constitute an essential dimension and a crucial element of communication in nursing education and in healthcare teams.

## RESUMEN

**Objetivos:**

comprender las percepciones de los estudiantes de enfermería sobre la comunicación no verbal utilizando la cronémica en el entorno quirúrgico.

**Métodos:**

estudio cualitativo, descriptivo-exploratorio, basado en la teoría de la comunicación no verbal de Edward T. Hall. Participaron 30 estudiantes de enfermería de una institución pública del Norte de Brasil. Los datos se recopilaron en mayo y junio de 2025 mediante entrevistas semiestructuradas, y se analizaron mediante análisis de contenido temático.

**Resultados:**

la cronémica se percibió como un elemento central en la dinámica del entorno quirúrgico. El ritmo acelerado generó extrañeza, ansiedad e inseguridad, lo que dificultó el aprendizaje. El período de espera reveló las prioridades del equipo, indicando quién recibe atención en el flujo de atención. La gestión del tiempo del personal de enfermería puso de relieve el conflicto entre la eficiencia de las tareas y la disponibilidad para la docencia, estableciéndose como un modelo formativo.

**Consideraciones Finales:**

la cronémica constituye una dimensión esencial y un elemento crucial de la comunicación en la formación en enfermería y en los equipos de atención sanitaria.

## INTRODUCTION

Initially, it is important to contextualize that, in the field of nursing, nonverbal communication is permeated by intersubjective dimensions such as time, movement, setting, encounter, and bodies. When considered in the context of teaching and learning in hospital practice, chronemics - i.e., communication related to temporal dynamics - can become essential to nurses’ practice in surgical settings^(1)^.

Specifically, chronemics communication considers how people perceive, react to, and use time in human interaction. This encompasses how they organize themselves around tasks and deadlines, speech speed, and the time spent listening, relating these temporal aspects to biological, psychological, and social dimensions^(1,2)^. Furthermore, this modality can manifest itself through punctuality, conversation duration, or waiting for service/procedure, the pace of activities, pauses, and even time orientation, conveying powerful messages implicit in the context^(2)^.

In the field of chronemics, there are two types of orientations regarding the temporal aspect: monochronic (M-time) and polychronic (P-time), both influenced by the setting or the culture itself^(3)^. M-time refers to completing an activity or function within a specific period, requiring concentration and responsibility to meet deadlines and schedules. In contrast, P-time refers to the dynamics of time a person takes to accomplish several things in the same period, prioritizing social relationships over completing a task^(3)^.

Although often underestimated in hospital settings, particularly in complex and dynamic contexts, chronemics is relevant because it complements or sometimes contradicts the spoken message, altering emotions, attitudes, and intentions, as well as interactions between students, professors, professionals, patients, and their families^(2)^.

Especially in surgical settings, characterized by high techno-assistance density, tension, controlled temperature, precision, and teamwork, duration management and perception take on even more intense meanings for students^(1,4)^. In this scenario, temporal dynamics cease to be merely an organizational resource, and begin to act as a communicational element for students^(5)^. Elements such as response speed, patient preparation duration, and procedure length can express organization, efficiency, respect, and even aspects related to prognosis^(1,4)^. The waiting time for tests or materials, and the pace of surgery, can also indicate priorities.

The way the surgical team manages the process and students’ experience in this setting significantly influences learning, skills development, work dynamics, organizational climate, and patient safety^(3)^. Furthermore, students’ entry into this context represents a moment of great anxiety and challenge^(6)^. Thus, the communication implicit over time, used and transmitted by the surgical team, shapes students’ perception of perioperative care, safety, and nurses’ leading role, constituting an essential training resource^(5)^.

Since it is a setting where verbal communication is limited by noise and the use of masks, there is an emphasis on focusing on and decoding nonverbal signals. In this context, the conscious use of nonverbal communication becomes fundamental for student-nursing team integration, understanding the setting dynamics, and developing competencies. Students are identified by professionals through observation of their posture, position, pace, and body movements adopted in the surgical setting^(3)^. This set of nonverbal elements reinforces the importance of including the teaching of nonverbal communication in nursing education.

However, there is little research on chronemics and, even more rarely, on the perception of students who learn surgical skills^(6)^. Factors such as institutional culture, prior experience, anxiety level, role clarity, and quality of pedagogical supervision can influence a student’s ability to perceive and interpret temporal cues in a surgical context^(4)^.

The relevance of investigating this phenomenon lies in improving nursing education. Furthermore, it offers the opportunity to make students more aware and skilled in interpreting nonverbal communication, including temporal dynamics, especially to promote effective and safe communication in surgical settings. Understanding students’ perception in this context can support innovative pedagogical strategies and sensitize surgical teams to the communicative impact of their time management^(3)^.

Valuing the learning of chronemics and the perception of pace, pauses, and waiting periods makes it possible to identify the needs of students, patients, and surgical team staff, explore opportunities, and assign important meanings to care, impacting the quality of the interprofessional relationship that they observe and begin to build^(2)^.

## OBJECTIVES

To understand nursing students’ perceptions of nonverbal communication and chronemics in surgical settings.

## METHODS

### Ethical aspects

The study was conducted in accordance with national and international ethical guidelines. It was approved by the *Universidade Federal de Roraima* Research Ethics Committee, under Opinion 7,531,201. All participants were informed about the study objectives, procedures, risks, and benefits, and signed the Informed Consent Form before data collection. Anonymity was guaranteed by coding the transcribed and analyzed statements, identified by the letter “I” and by an increasing numerical sequence of participation (I1, I2... I30).

### Study design

This is a qualitative, descriptive-exploratory study, written in accordance with Consolidated criteria for Reporting Qualitative research international guidelines^(7)^.

### Study setting

The study was conducted in a classroom at a public higher education institution located in the northern Brazil. This setting was chosen because of the need for a reflective and protected setting that would allow students to revisit and deepen their experiences in surgical settings. By physically distancing them from this context, the aim was to minimize hierarchical, emotional, and operational influences, allowing their perceptions to be revealed freely and consciously, an essential condition for understanding the phenomenon under investigation.

### Data source

The study population consisted of students regularly enrolled in the undergraduate nursing program at the aforementioned institution, who had already completed or were currently taking the course with practical components in a surgical setting. The social group was selected by convenience, based on invitations made in the classroom to students who met the inclusion criteria. The exclusion criteria were working in an operating room as a nursing technician and lack of schedule availability.

Initially, 40 students who had gone through this experience in recent semesters were invited. Of these, 35 accepted the invitation. However, two were excluded because they worked as nursing technicians in surgical centers, and three dropped out after the initial acceptance due to scheduling conflicts. Thus, the final number of participants was 30 students.

Access for participants was granted after authorization from the course coordinator. The principal investigator, a faculty member of the institution, presented the study in classrooms during eligible periods, explaining the objectives and procedures, and inviting interested students to participate. Interviews were scheduled individually at times convenient for students.

To mitigate biases related to the researcher’s dual role, the interviews were conducted in a neutral setting, detached from any evaluative activity. Although the researcher did not work directly with participants nor was involved in related clinical disciplines, confidentiality, anonymity, autonomy, and voluntariness were guaranteed throughout the process, in order to foster a safe space for the free and authentic expression of their perceptions. The time between the end of clinical practice in surgical settings and the interview ranged from three to six weeks, with a mean of 4.5 weeks.

### Data collection and organization

Data collection took place between May and June 2025, through individual semi-structured interviews. The procedure was conducted by a single researcher, who holds a doctoral degree in nursing, with expertise in studies on nonverbal communication and a qualitative approach, in a private room at the university, ensuring privacy and comfort for participants.

The total number of interviews was determined by the criterion of theoretical saturation, identified from the 28^th^ meeting onwards, with the 30^th^ session concluding to confirm information stability and recurrence. This entire stage was recorded with students’ prior consent, using an audio recorder on a telephone. The mean duration of each interview was 45 minutes.

The interview script contained open-ended questions that explored students’ perception of the temporal dynamics in surgical settings (e.g.,“How do you perceive the pace of work of the team in a surgical setting?”, “Did you feel that the temporal dynamics of waiting for something communicated any message?”, “How did the nursing staff’s time management influence your learning or interaction?”). The closed-ended questions, in turn, aimed to collect sociodemographic data, such as students’ age and gender, course period, and length of practice in a surgical setting. This data was presented in absolute and percentage terms. To refine the script and approach, a pilot test was conducted with two students, who were not included in the final sample.

In order to ensure study rigor and credibility, the researcher employed reflexivity, as recommended by qualitative research guidelines. This practice was communicated to students, constituting an essential strategy to reduce possible responses that professors’ position might induce.Furthermore, the use of a semi-structured script and the application of a systematized technique for data analysis reinforced the objectivity of the interpretive process.

To ensure analysis validity and reliability, independent double coding of a set of interviews was carried out, followed by intersubjective validation of the categories by two researchers experienced in qualitative research. Any discrepancies were discussed until an interpretative consensus was reached, ensuring greater methodological reliability and theoretical consistency of the emerging categories.

### Data analysis

Quantitative sociodemographic data processing was guided by descriptive statistics, using absolute (N) and relative (%) frequencies and measures of central tendency to characterize the participants. The presentation of these data followed criteria of clarity and standardization, allowing for a detailed description of the sample regarding age, sex, course period, and length of practice in a surgical setting.

For qualitative data, thematic content analysis was adopted^(8)^, a systematic technique for identifying, analyzing and presenting themes in qualitative data, following its three chronological stages: 1) Pre-analysis: material text skimming, initial organization and formulation of hypotheses and objectives; 2) Material exploration: data coding, transforming the raw data into units of registration and classification to form emerging thematic categories; 3) Processing of results, inference and interpretation: reflective analysis of the identified thematic categories, relating them to the theoretical framework, the study objective and relevant literature.

To organize, code and manage qualitative data, the Delve is intuitive AI assisted qualitative analysis version 2024^(9)^ software was used, a technological resource that allows access to textual data, assign codes and identify themes through interactive panels and exportable reports, facilitating the identification of patterns and the construction of thematic categories.

## RESULTS

The sample consisted of students aged between 18 and 32 years, with (n=8; 26.7%) aged 18 to 22 years, (n=15; 50%) aged 23 to 27 years, and (n=7; 23.3%) aged 28 to 32 years. Females predominated (n=24; 80%). Concerning the course period, most were in their 8^th^ or 9^th^ semester (n=18; 60%). All participants had completed surgical practice, either as an internship or technical visit, for a period ranging from three to six weeks. Practice time distribution showed that (n=9; 30%) had between three and four weeks of experience, while (n=21; 70%) had between five and six weeks.

Based on the analysis of statements, three central thematic categories emerged, which express nursing students’ perception of nonverbal communication using chronemics in surgical settings.

The first thematic category, entitled “Surgical pace as a reflection of team interaction”, encompasses students’ perceptions of the cadence of actions and timepace, and how this pace influences team coordination and individual anxiety levels. Participants understood surgical settings as a space where temporal dynamics are a determining factor, dictating the slowness and speed of procedures, as well as the coordination among the professionals on the surgical team, as observed below:

*When I started, everything was new. Everything happened very quickly, without interruptions. If someone asked me to do something outside of what I was already doing, I felt very nervous.* (I3)

*You can tell when things are getting hectic by the pace. Everyone starts moving faster, talking less. The messenger seems to fly around looking for supplies. That made me tense and anxious, thinking something was going wrong.* (I14)

*Some surgeries flow smoothly at a good pace,“it’s like a dance”. In others, the pace is broken, one patient waits for the next, it feels like something is missing. This communicates disorganization.* (I21)

*The scrub nurse passing the instruments to the surgeon sometimes does it so quickly that I can’t even keep up. That shows their experience, but for those of us who are learning, it’s a little daunting.* (I5)

The ability to keep up with or anticipate the surgical pace was frequently associated with a sense of belonging and a perception of competence. Furthermore, feeling in sync with the timing of activities reinforced students’ integration into the team dynamics. On the other hand, difficulty adapting to the speed imposed by the procedure or the team generated feelings of inadequacy and anxiety.

In the second thematic category, entitled “Waiting as an expression of priority, tension, or disorganization”, students attributed diverse meanings to the intervals experienced in surgical settings, whether for the arrival of patients, the team, or for specific procedures. These pauses, although seemingly passive, were interpreted as forms of nonverbal communication, conveying messages about priority, organization, and, in some cases, disregard.

The wait duration and nature could indicate the urgency of a situation, the efficiency of the planning, or a lack of coordination. For students, the period of silent inactivity became a time of observation, interpretation, and translation of signals, influencing their understanding of the care dynamics and the importance of temporal aspects in workflow management. These understandings can be evidenced in the statements presented below:

*Seeing a patient waiting in the hallway for a long time sometimes conveys that they are not a priority at that moment or that the room is running late. It gives a bad feeling.* (I2)

*When the anesthesiologist is late, everything is delayed. They report a problem with the surgical schedule or perhaps that he was dealing with an emergency, but nobody explains.* (I15)

*There was one time when we waited a very long time for a specific material. The silence in the room, the waiting, conveyed tension, worry about whether the material would arrive on time.* (I11)

*On the other hand, when everything happens on time-the induction of anesthesia, the start of surgery-it communicates efficiency and organization. It gives security to us who are in the teaching-learning process, as well as to the patient.* (I9)

Thus, waiting is not perceived merely as a lapse of time, but as a powerful indicator of organizational culture and quality of care planning in surgical settings.

The third thematic category, entitled “Time management by the nursing team as a care model and influence on student learning”, explores how these professionals manage their work in surgical settings and how this time management serves as a model for students, directly impacting their learning and perception of care.

Students reported that the way nurses and nursing technicians organized their time and prioritized tasks communicated professionalism or disorganization, influencing safety perception in teaching settings. Professionals who demonstrated clear time management, performing pre-operative checks without rushing and managing the flow of materials proactively, were seen as models of excellence, as illustrated in the following statements:

*The circulating nurse knows what will be needed even before the surgeon asks. This shows experience and that he values time: ours and the patient’s. He was able to explain things to me during the breaks.* (I30)

*When a nurse or technician is organized and demonstrates that they have time to answer a question, even if it’s just for a minute, it communicates that learning is important.* (I19)

*The lack of time, the rushed pace, or the nurse’s impatience when we asked a question communicated that we were a problem, that we were there to get in the way.* (I7)

*This rush to communicate and the time it takes to speak with us* [student] *generates anxiety and inhibits our search for clarification. This directly affects learning.* (I25)

It is thus clear that nurses’ time, when well managed and dedicated to interaction with students, becomes a valuable pedagogical resource, reinforcing the importance of professionals as a facilitator of learning. This time management allows students to observe, understand, and reflect on care practices, promoting more meaningful learning integrated into the context of care.

## DISCUSSION

In light of the findings, discussions were directed toward deepening the Edward T. Hall theoretical framework^(3)^, especially M-time and P-time orientations. In this context, the surgical setting, predominantly M-time, is characterized by rigid deadlines, linearity, and a focus on surgery, requiring precise temporal coordination, where deviations or interruptions are perceived as breaks in the flow. However, students from more P-time contexts perceived this temporal dynamic as a sensitive communicational element, capable of influencing both learning and interaction with the team.

This perception transcends the chronological dimension, configuring itself as a form of nonverbal communication that expresses pace, expectations, and modes of coexistence in healthcare work. Within the context of the tension between procedure time and learning pace, thematic categories are inserted, detailing how this temporal management manifests itself in students’ experience. Furthermore, the perception of temporal adequacy reinforced students’ sense of belonging. The analysis of these perceptions thus highlights the profound chronemic tension inherent in teaching-learning settings.

The first thematic category, “Surgical pace as a reflection of team interaction”, highlights that students perceived surgical settings as a space characterized by a fast pace, continuous focus, and few pauses, which generated feelings of strangeness, anxiety, and insecurity during adaptation to the setting^(10)^. At times, they reported feeling a “broken pace”, when simultaneous demands disrupted the team’s expected workflow.

These reports reinforce that the surgical pace, in addition to indicating efficiency, functions as a communicator of expectations and an emotional regulator in the training process, aligning with the M-time perspective and P-time intrusions that characterize highly complex care settings.

The contrast between M-time and P-time represents the core of students’ perception of chronemics. While the surgical setting demands speed and coordination, students need relational and reflective time, typical of P-time, to understand the context and consolidate learning. Consequently, the “broken pace” can be understood as a P-time intrusion into M-time, i.e., an interruption caused by simultaneous demands that overlap the team’s expected cadence^(3,6)^.

From a pedagogical standpoint, professors and mentors must mediate this tension, interpreting the team’s temporal deviation not as a sign of chaos, but as an educational resource to be analyzed, guiding students to interpret these “breaks” as critical moments for observation and adaptation.

The thematic category “Waiting as an expression of priority, tension, or disorganization” highlights that, in a M-time culture, prolonged waiting, when not explained or justified, communicates negligence or lack of priority, generating insecurity and a negative perception of the service organization^(3,8)^. “The wait is primarily perceived” by students as a failure in the efficient execution of M-time, but it often results from unmanaged P-time demands, indicating the need for intentional team communication.

This study demonstrates that chronemics is not merely a logistical backdrop, but an autonomous and crucial nonverbal dimension that communicates priority, status, and competence. The anxieties reported by students stem not only from eye contact or inadequate physical distance (kinesics and proxemics), but also from accelerated pace or unjustified waiting time. In this way, this research fills a gap by focusing on the training and communicational power of time, often invisible in health communication analyses.

The intentional use of P-time, when justifying delays and guiding the student, can transform waiting time into observation time, reducing misinterpretations and strengthening the pedagogical bond^(1)^. This finding reinforces the importance of employing P-time strategically to explain M-time and mitigate negative interpretations, ensuring that waiting is experienced as a time of learning, not exclusion^(10)^.

For students, this temporal structure represents an adaptation challenge, as it demands learning an intense work pace, supported by rigid protocols and the need for quick and coordinated responses. Consequently, continuous observation of professional models and practical experience favor the development of the ability to interpret chronemic signals and adjust one’s own pace, improving communication skills^(11)^.

Influenced by the temporal dynamics of the surgical setting, marked by pace, pauses, and waiting periods, participants developed communication, relational, and time self-management skills, demonstrating progressive adaptation to the M-time context, in which time becomes a language and a training instrument.

In this context, students are confronted with a temporal culture different from other teaching scenarios, being led to develop skills of attention, agility, and integration into the multidisciplinary team, without losing sight of the human aspect of care^(12)^. Furthermore, the teaching-learning process in a surgical setting can be associated with P-time, characterized by the simultaneous and flexible experience of time, interpersonal relationships, empathy, and the ability to adapt to the context^(1)^.

This dimension manifests itself when the student begins to realize that, beyond simply completing tasks, it is necessary to recognize human pace, the team’s time in its cooperative dynamics, and their own internal time for learning and professional development^(10)^.

In this regard, the thematic category “Time management by the nursing team as a model of care and influence on learning” reinforces the evidence of the conflict between M-time and P-time, which directly reflects the tension between productivity and relational availability. The way professionals allocate time to teaching communicates values, respect, commitment, and inclusion^(3,12)^. On the other hand, unavailability and haste convey, in a nonverbal way, the exclusion of students from the care dynamic, compromising their learning^(13)^.

The findings reinforce the nurses’ role as a mediator of chronemics, responsible for balancing the efficiency of M-time with the relational availability of P-time, recognizing time as a pedagogical and communicational resource. This stance communicates a powerful message: productivity should not negate the need for humanized teaching and care, transforming time management into a training, inclusive leadership model.

Furthermore, students are challenged to integrate the institutional pace with relational time, marked by pauses, listening, and sensitivity. In short, learning in a surgical setting implies not only mastering techniques and workflows, but also articulating different temporal perceptions, promoting safer and more conscious practical learning^(11)^.

Nurses, operating within a logic of precision, must simultaneously adopt a relational stance that highlights the challenge of time management in pedagogical practice, where pace ceases to be merely a tool and begins to function as a language^(3)^. National and international studies point to the relevance of chronemics in professional communication, although it is less explored than other nonverbal dimensions, such as kinesics or proxemics^(14-16)^.

Students’ perceptions of the influence of chronemics on their anxiety and learning reinforce the need to explicitly incorporate this topic into nursing curricula, using active methodologies. The conflict between M-time and P-time directly contributes to this pedagogical practice by providing support for reflective training. Furthermore, it prepares students to act ethically in high-pressure and fast-paced contexts^(6)^. Strategies such as high-fidelity clinical simulation, structured debriefing sessions, and reflective analysis promote the development of conscious communication skills^(6,17)^.

The pace, pause, and waiting dimensions are configured as significant pedagogical and care resources that favor the identification of the needs of students and patients, positively impacting quality of care and professional training^(8)^. Raising team awareness about the impact of time management on communication and adopting simple practices, such as justifying delays or providing opportunities for guidance, helps reduce anxiety triggers and strengthen bonds^(5,18)^.

When understood in depth, chronemics contribute to the training of nurses who are sensitive, empathetic, critical, and attentive to the subjective dimensions of care^(4,18)^. The experience of time spent in surgical settings influences students’ confidence, decision-making, and professional identity, reinforcing the importance of training settings that promote reflection on time management^(4,19)^.

In high-pressure settings, proper time management promotes more humane care and strengthens interpersonal relationships, enhancing dialogue, empathy, patient safety, and student satisfaction^(2,4)^. Active listening, as a temporal experience, can reveal organizational gaps in the surgical setting that, if corrected, enhance teaching and multidisciplinary integration^(6,20)^.

In this context, nonverbal temporal communication should constitute a strategic focus in the training of nurse leaders, capable of planning care and organizing the team’s time^(8,21)^. By promoting balanced time management, these leaders contribute to stress reduction, increased role clarity, improved quality and safety of care, and strengthened pedagogical development^(7)^.

Incorporating chronemics into interpersonal and communication skills training can enhance temporal awareness, improve coordination of actions and responses to critical situations, and contribute to reducing errors and accidents^(6,21)^. Raising awareness of chronemics among future nurses deepens their understanding of care and strengthens the human dimension of their training, preparing professionals capable of integrating technical efficiency, sensitivity, and communication skills in complex contexts^(8)^.

### Study limitations

Limitations of this study include the scope of data collection, which was limited to nursing students from a single public institution in northern Brazil, thus restricting the generalizability of the findings. Additionally, the fact that data collection took place in the classroom and relied on participants’ retrospective memories resulted in a time gap, albeit short, between the end of surgical practice and the interviews. This may have influenced the accuracy of the recollections and interpretations of chronemics, especially since this study focused on perceptions of time and pace.

Another limitation lies in the potential influence of the researcher, who is a faculty member at the institution. Despite the mitigation strategies adopted, it is recognized that the hierarchical professor-student relationship may have generated socially desirable responses, configuring a bias of social desirability and credibility. Furthermore, even with the relatively short gap, it is possible that students’ memories were influenced by the interval between the practice and the interview, constituting a study limitation. On the other hand, the detailed description of the method and context contributed to the transparency of the methodological process, allowing for an assessment of the transferability of the findings to similar realities.

### Contributions to nursing, health, or public policy

This study incorporates essential understandings into the field of nursing care by placing a fundamental tool at the center of the debate: communication. Another relevant point is to give students a voice regarding a little-explored aspect of nonverbal communication in surgical settings: the importance of chronemics for their nursing training and practice.

The findings suggest the need to include this topic more explicitly in nursing curricula and to raise awareness among clinical teams about the communicative impact of self-management of time and pace in surgical settings. Clarity in the meaning of pauses, movements, and execution times can foster a safer, more organized, and welcoming teaching and work setting for future nurses.

Nevertheless, the results offer a significant contribution by highlighting the temporal dimension of nonverbal communication in nursing education, as well as by discussing its pedagogical and care implications, and by broadening the understanding of how time management influences the teaching-learning process and quality of care.

## FINAL CONSIDERATIONS

Understanding time, in its various manifestations of pace, waiting, duration, and management, presents itself as a significant communicational element for nursing care. Participants perceived chronemics as an indicator of the surgical team dynamics, service (dis)organization, care priorities, and availability for interaction and teaching-learning, which can influence the production of some degree of anxiety in the training experience in surgical settings.

Therefore, it is recommended to develop high-fidelity clinical simulations focusing on time management and structured debriefing for reflection on pace and pauses. Furthermore, it is suggested to create temporal communication protocols for surgical teams, encouraging the justification of delays and the provision of intentional “pause minutes” to guide students, improve their communication skills and competencies, and enhance the fluidity of perioperative care.

Recognizing chronemics as an essential pedagogical resource strengthens the humanistic and communicational training of future nurses, enabling them to balance technical efficiency with sensitivity and patient-centered care, even in highly complex settings.

## Data Availability

The research data are available only upon request.
