# Supplementary material for: Nursing students’ perceptions of nonverbal communication using chronemics in surgical settings
Source: Rev Bras Enferm. 2026 Sep 28;79:e20250359. doi: 10.1590/0034-7167-2025-0359 (PMC13623387; doi:10.1590/0034-7167-2025-0359)
Supplement: Translation

# Translation: Percepções de estudantes de enfermagem sobre comunicação não verbal cronêmica no ambiente cirúrgico

**DOI:** 10.1590/0034-7167-2025-0359pt

**Authors:** Albert Lengruber de Azevedo, Sílvia Teresa Carvalho de Araujo, Paulo Sergio da Silva, Francisco Gleidson de Azevedo Gonçalves

**Affiliations:** I Universidade Federal de Roraima. Boa Vista, Roraima, Brasil; II Universidade Federal do Rio de Janeiro. Rio de Janeiro, Rio de Janeiro, Brasil; III Universidade Federal de Lavras. Lavras, Minas Gerais, Brasil; IV Instituto Estadual de Hematologia Arthur de Siqueira Cavalcanti. Rio de Janeiro, Rio de Janeiro, Brasil

**Keywords:** Percepção, Estudantes de Enfermagem, Comunicação Não Verbal, Centro Cirúrgico Hospitalar, Educação em Enfermagem.

## Abstract

**Objetivos:**

compreender a percepção de estudantes de enfermagem sobre comunicação não verbal usando cronêmica no ambiente cirúrgico.

**Métodos:**

estudo qualitativo, descritivo-exploratório, fundamentado na teoria da comunicação não verbal de Edward T. Hall. Participaram 30 estudantes de enfermagem de instituição pública da região Norte do Brasil. Os dados foram coletados em maio e junho de 2025, a partir de entrevistas semiestruturadas, e analisados segundo a análise de conteúdo temática.

**Resultados:**

a cronêmica foi percebida como elemento central na dinâmica do ambiente cirúrgico. O ritmo acelerado gerou estranhamento, ansiedade e insegurança, dificultando a aprendizagem. A espera revelou prioridades da equipe, indicando quem recebe atenção no fluxo assistencial. A gestão do tempo pela enfermagem evidenciou o conflito entre eficiência das tarefas e disponibilidade para o ensino, configurando-se como modelo formativo.

**Considerações Finais:**

a cronêmica constitui uma dimensão essencial e um elemento crucial da comunicação na formação em enfermagem e nas equipes de saúde.

## INTRODUÇÃO

Inicialmente, é oportuno contextualizar que, no campo da enfermagem, a comunicação não verbal é atravessada por dimensões intersubjetivas, como tempo, movimento, ambiente, encontro e corpos. Quando considerada no contexto do ensino-aprendizagem da prática hospitalar, a cronêmica - isto é, aquela relacionada à dinâmica temporal - pode se tornar essencial ao exercício do enfermeiro no ambiente cirúrgico^(1)^.

Especificamente, a comunicação cronêmica considera a forma como as pessoas percebem, reagem e utilizam o tempo na interação humana. Isso engloba a maneira como se organizam diante de tarefas e prazos, a velocidade da fala e o tempo dedicado à escuta, relacionando esses aspectos temporais às dimensões biológicas, psicológicas e sociais^(1,2)^. Além disso, essa modalidade pode se manifestar pela pontualidade, pela duração de uma conversa, ou pela espera por um atendimento/procedimento, ritmo das atividades, pausas e até mesmo pela orientação temporal, transmitindo mensagens poderosas implícitas ao contexto^(2)^.

No campo da cronêmica, existem dois tipos de orientações sobre o aspecto temporal, a monocrônica (*M-time*) e a policrônica (*P-time*), ambas influenciadas pelo ambiente ou pela própria cultura^(3)^. A *M-time* diz respeito à realização de uma atividade ou função em um determinado período, requerendo concentração e responsabilidades para o cumprimento dos prazos e horários. Em contraste, *P-time* refere-se à dinâmica do tempo que uma pessoa leva para executar várias coisas em um mesmo período, havendo uma priorização entre manter relações sociais e concluir uma tarefa^(3)^.

Embora frequentemente subestimada nos serviços hospitalares, principalmente em contextos complexos e dinâmicos, a cronêmica é relevante por complementar ou, por vezes, contradizer a mensagem falada, alterando emoções, atitudes e intenções, além das interações entre estudantes, professores, profissionais, pacientes e seus familiares^(2)^.

Especialmente no ambiente cirúrgico, caracterizado pela alta densidade tecno-assistencial, tensão, temperatura controlada, precisão e trabalho em equipe, a gestão e a percepção da duração assumem significados ainda mais intensos para os estudantes^(1,4)^. Nesse cenário, a dinâmica temporal deixa de ser apenas um recurso organizacional, passando a atuar como elemento comunicacional para aquele que aprende^(5)^. Elementos como a rapidez das respostas, a duração do preparo do paciente e o período dos procedimentos podem expressar organização, eficiência, respeito e até mesmo aspectos relacionados ao prognóstico^(1,4)^. A espera por exames ou materiais e a cadência da cirurgia podem, também, comunicar prioridades.

O modo como a equipe cirúrgica gerencia o processo e a experiência do estudante nesse ambiente influencia significativamente a aprendizagem, o desenvolvimento de competências, a dinâmica de trabalho, o clima organizacional e a segurança do paciente^(3)^. Além disso, a entrada do estudante nesse contexto representa um momento de grande ansiedade e desafio^(6)^. Assim, a comunicação implícita pelo tempo, utilizada e transmitida pela equipe cirúrgica, molda a percepção do estudante sobre a assistência perioperatória, a segurança e o protagonismo do enfermeiro, constituindo-se como recurso formativo essencial^(5)^.

Por ser um ambiente em que a comunicação verbal é limitada pelo ruído e pelo uso de máscaras, há ênfase no foco e na decodificação de sinais não verbais. Nessa condição, o uso consciente da comunicação não verbal se torna fundamental para a integração estudante-equipe de enfermagem, a compreensão da dinâmica do ambiente e o desenvolvimento de competências. Estudantes são identificados pelos profissionais por meio de observação da postura, posição, ritmo e movimento corporal adotados no ambiente cirúrgico^(3)^. Esse conjunto de elementos não verbais reforça a importância de incluir o ensino da comunicação não verbal na formação em enfermagem.

No entanto, há poucas pesquisas sobre a cronêmica e, ainda mais raramente, sobre a percepção do estudante que aprende competências cirúrgicas^(6)^. Elementos como cultura institucional, experiência prévia, nível de ansiedade, clareza dos papéis e qualidade da supervisão pedagógica podem influenciar a capacidade do estudante de perceber e interpretar sinais temporais no contexto cirúrgico^(4)^.

A relevância de investigar esse fenômeno reside na melhoria da formação em enfermagem. Além disso, oferece a oportunidade de tornar os estudantes mais conscientes e hábeis para a interpretação da comunicação não verbal, incluindo a dinâmica temporal, sobretudo para promover uma comunicação eficaz e segura no ambiente cirúrgico. Compreender a percepção do estudante, nesse contexto, pode subsidiar estratégias pedagógicas inovadoras e sensibilizar as equipes cirúrgicas para o impacto comunicacional da sua gestão do tempo^(3)^.

Valorizar a aprendizagem da cronêmica e a percepção do ritmo, das pausas e das esperas possibilita identificar necessidades do estudante, do paciente e dos membros da equipe cirúrgica, explorar oportunidades e atribuir significados importantes ao cuidado, impactando a qualidade da relação interprofissional que ele observa e começa a construir^(2)^.

## OBJETIVOS

Compreender a percepção de estudantes de enfermagem sobre comunicação não verbal cronêmica no ambiente cirúrgico.

## MÉTODOS

### Aspectos éticos

O estudo foi conduzido conforme diretrizes éticas nacionais e internacionais. Foi aprovado pelo Comitê de Ética em Pesquisa da Universidade Federal de Roraima, sob Parecer n^o^ 7.531.201. Todos os participantes foram informados sobre os objetivos, procedimentos, riscos e benefícios do estudo, e assinaram o Termo de Consentimento Livre e Esclarecido antes da coleta de dados. O anonimato foi garantido pela codificação dos depoimentos transcritos e analisados, identificados pela letra “E” e por uma sequência numérica crescente de participação (E1, E2... E30).

### Desenho do estudo

Estudo qualitativo, descritivo-exploratório, redigido de acordo com o guia internacional *Consolidated criteria for Reporting Qualitative research* ^(7)^.

### Cenário do estudo

O estudo foi realizado em sala de aula de uma instituição de ensino superior pública localizada na região Norte do Brasil. A escolha desse cenário decorre da necessidade de um ambiente reflexivo e protegido que favorecesse aos estudantes a retomada e o aprofundamento das vivências no ambiente cirúrgico. Ao afastá-los fisicamente desse contexto, buscou-se minimizar influências hierárquicas, emocionais e operacionais, permitindo que suas percepções fossem reveladas de forma livre e consciente, condição essencial para a apreensão do fenômeno investigado.

### Fonte de dados

A população do estudo foi composta por estudantes regularmente matriculados no curso de graduação em enfermagem da referida instituição, que já haviam cursado ou estavam cursando a disciplina com componentes práticos em ambiente cirúrgico. O grupo social foi selecionado por conveniência, a partir de convite realizado em sala de aula aos estudantes que atendiam aos critérios de inclusão. Os critérios de exclusão foram trabalhar em centro cirúrgico como técnico de enfermagem e indisponibilidade de horário.

Inicialmente, foram convidados 40 estudantes que haviam passado por essa experiência nos últimos semestres. Desses, 35 aceitaram o convite. No entanto, dois foram excluídos por trabalharem como técnico de enfermagem em centro cirúrgico, e três desistiram após o aceite inicial por indisponibilidade de horário. Assim, o número final de participantes foi de 30 estudantes.

O acesso aos participantes foi permitido após autorização da coordenação do curso. O pesquisador principal, docente do curso da instituição de ensino, apresentou o estudo em salas de aula dos períodos elegíveis, explicando os objetivos e procedimentos, e convidando os interessados a participar. As entrevistas foram agendadas individualmente em horários convenientes para os estudantes.

Para mitigar os vieses relacionados à dupla função do pesquisador, as entrevistas foram conduzidas em ambiente neutro, desvinculado de qualquer atividade avaliativa. Embora não tenha atuado diretamente com os participantes nem tenha se envolvido nas disciplinas clínicas relacionadas, reforçou-se a garantia de confidencialidade e anonimato, a autonomia e a voluntariedade durante todo o processo, de modo a favorecer um espaço seguro para a expressão livre e autêntica de suas percepções. O tempo entre o término da prática clínica no ambiente cirúrgico e a realização da entrevista variou de três a seis semanas, com uma média de 4,5 semanas.

### Coleta e organização dos dados

A coleta de dados ocorreu entre os meses de maio e junho de 2025, a partir de entrevista semiestruturada individual. O procedimento foi conduzido por um único pesquisador, que é doutor em enfermagem, com *expertise* em estudos sobre comunicação não verbal e abordagem qualitativa, em uma sala reservada na universidade, garantindo a privacidade e o conforto necessário dos participantes.

O número total de entrevistas foi definido pelo critério de saturação teórica, identificada a partir do 28º encontro, com o encerramento na 30ª sessão para confirmar a estabilidade e a recorrência das informações. Toda essa etapa foi gravada com consentimento prévio do estudante, a partir de gravador de áudio em aparelho telefônico. O tempo médio de cada entrevista foi de 45 minutos.

O roteiro de entrevista continha perguntas abertas que exploravam a percepção do estudante sobre a dinâmica temporal no ambiente cirúrgico (exemplo: “Como você percebe o ritmo de trabalho da equipe em ambiente cirúrgico?”, “Você sentiu que a dinâmica temporal de espera por algo comunicou alguma mensagem?”, “Como a gestão do tempo pela enfermagem influenciou sua aprendizagem ou interação?”). As perguntas fechadas, por sua vez, visavam coletar dados sociodemográficos, como idade e sexo dos estudantes, período do curso, e tempo de prática em ambiente cirúrgico. Esses dados foram apresentados de forma absoluta e percentual. Para refinar o roteiro e a abordagem, foi realizado teste piloto com dois estudantes, que não foram incluídos na amostra final.

Com intuito de assegurar o rigor da credibilidade do estudo, foi empreendida a reflexividade do pesquisador, conforme preconizam as diretrizes de pesquisa qualitativa. Essa foi uma prática comunicada aos estudantes, constituindo estratégia essencial para reduzir possíveis respostas que a posição de docente pudesse induzir. Além disso, a utilização de roteiro semiestruturado e a aplicação de técnica sistematizada para análise dos dados reforçaram a objetividade no processo interpretativo.

Para garantir a validade e a fidedignidade da análise, realizou-se a dupla codificação independente de um conjunto de entrevistas, seguida por validação intersubjetiva das categorias por dois pesquisadores experientes em pesquisa qualitativa. As eventuais divergências foram discutidas até o estabelecimento de consenso interpretativo, assegurando maior confiabilidade metodológica e consistência teórica das categorias emergentes.

### Análise dos dados

O tratamento dos dados sociodemográficos de natureza quantitativa foi orientado por estatística descritiva, utilizando frequências absolutas (N), relativas (%) e medidas de tendência central para caracterização dos participantes. A apresentação desses dados seguiu critérios de clareza e padronização, permitindo a descrição detalhada da amostra quanto à idade, sexo, período do curso e tempo de prática em ambiente cirúrgico.

Para os dados qualitativos, foi adotada a análise de conteúdo temática^(8)^, uma técnica sistemática para identificar, analisar e apresentar temas nos dados qualitativos, seguindo suas três etapas cronológicas: 1) Pré-análise: leitura flutuante do material, organização inicial e formulação de hipóteses e objetivos; 2) Exploração do material: codificação dos dados, transformando os dados brutos em unidades de registro e classificação para formar categorias temáticas emergentes; 3) Tratamento dos resultados, inferência e interpretação: análise reflexiva das categorias temáticas identificadas, relacionando-as ao referencial teórico, ao objetivo do estudo e à literatura pertinente.

Para organizar, codificar e gerenciar os dados qualitativos, utilizou-se o *software Delve - an*

*intuitive AI-assisted qualitative analysis -,* versão 2024^(9)^, um recurso tecnológico que permite dados textuais, atribuir códigos e identificar temas por meio de painéis interativos e relatórios exportáveis, facilitando a identificação de padrões e a construção das categorias temáticas.

## RESULTADOS

A amostra foi composta por estudantes com idades entre 18 e 32 anos, sendo (n=8; 26,7%) de 18 a 22 anos, (n=15; 50%) de 23 a 27 anos e (n=7; 23,3%) de 28 a 32 anos. Predominou o sexo feminino (n=24; 80%). Quanto ao período do curso, a maior parte encontrava-se entre o 8º e o 9º períodos (n=18; 60%). Todos os participantes haviam realizado prática em ambiente cirúrgico, seja em estágio ou visita técnica, por um período que variou de três a seis semanas. A distribuição do tempo de prática mostrou que (n=9; 30%) possuíam entre três e quatro semanas de experiência, enquanto (n=21; 70%) possuíam entre cinco e seis semanas.

A partir da análise dos depoimentos, emergiram três categorias temáticas centrais, que expressam a percepção dos estudantes de enfermagem acerca da comunicação não verbal cronêmica no ambiente cirúrgico.

A primeira categoria temática, intitulada “Ritmo cirúrgico como reflexo da interação da equipe”, abrange a percepção dos estudantes sobre a cadência das ações e o ritmo do tempo e como esse ritmo influencia a coordenação da equipe e o nível de ansiedade individual. Os participantes compreenderam o ambiente cirúrgico como um espaço no qual a dinâmica temporal é um fator determinante, ditando a lentidão e a velocidade dos procedimentos, bem como a coordenação entre os profissionais da equipe cirúrgica, conforme observado a seguir:

*Quando eu comecei, era tudo novo. Tudo o que acontecia era muito rápido, sem interrupções. Se alguém me pedisse pra fazer fora do que eu já estava fazendo, eu me sentia muito nervosa.* (E3)

*Você percebe quando a coisa aperta pelo ritmo. Todo mundo começa a se mover mais rápido, a falar menos. O circulante parece voar buscando material. Isso me deixava tensa e ansiosa, achando que algo estava dando errado.* (E14)

*Tem cirurgia que flui num ritmo bom: “parece uma dança”. Há outras em que o ritmo é quebrado, um espera o outro, parece que falta alguma coisa. Isso comunica desorganização*. (E21)

*O instrumentador passando os instrumentos para o cirurgião às vezes é tão rápido que eu nem conseguia acompanhar. Isso mostra a experiência deles, mas, pra gente que tá aprendendo, assusta um pouco*. (E5)

A capacidade de acompanhar ou antecipar o ritmo cirúrgico estava frequentemente associada à sensação de pertencimento e à percepção de competência. Além disso, sentir-se em sintonia com o tempo das atividades reforçou a integração dos estudantes à dinâmica da equipe. Por outro lado, a dificuldade em se adaptar à velocidade imposta pelo procedimento ou pela equipe gerou sentimentos de inadequação e ansiedade.

Na segunda categoria temática, intitulada “Espera como expressão de prioridade, tensão ou desorganização”, os estudantes atribuíram significados diversos aos intervalos vivenciados no ambiente cirúrgico, seja para chegada do paciente, da equipe ou para procedimentos específicos. Essas pausas, embora aparentemente passivas, foram interpretadas como formas de comunicação não verbal, transmitindo mensagens sobre prioridade, organização e, em alguns casos, descaso.

A duração e a natureza da espera podiam indicar a urgência de uma situação, a eficiência do planejamento ou a falta de coordenação. Para os estudantes, o período de inatividade silenciosa se tornou um momento de observação, interpretação e tradução de sinais, influenciando sua compreensão sobre a dinâmica do cuidado e a importância dos aspectos temporais na gestão do fluxo de trabalho. Essas compreensões podem ser evidenciadas nos depoimentos apresentados a seguir:

*Ver o paciente esperando no corredor por muito tempo às vezes comunica que ele não é a prioridade naquele momento ou que a sala está atrasada. Dá uma sensação ruim.* (E2)

*Quando o anestesista demora a chegar, atrasa tudo. Comunica uma falha na organização do mapa cirúrgico ou talvez que ele estava numa emergência, mas ninguém explica*. (E15)

*Teve uma vez que esperamos muito por um material específico. O silêncio na sala, a espera, comunicava tensão, preocupação se o material ia chegar a tempo*. (E11)

*Por outro lado, quando tudo acontece no tempo certo, a indução anestésica, o início da cirurgia, isso comunica eficiência e organização. Dá segurança para nós que estamos em processo ensino-aprendizagem, assim como para o paciente também*. (E9)

Desse modo, a espera não é percebida apenas como um lapso de tempo, mas como um poderoso sinalizador da cultura organizacional e da qualidade do planejamento assistencial no ambiente cirúrgico.

A terceira categoria temática, denominada “Gestão do tempo pela equipe de enfermagem como modelo de cuidado e influência no aprendizado discente”, explora como esses profissionais gerenciam o seu trabalho no ambiente cirúrgico e como essa gestão temporal serve de modelo para os estudantes, impactando diretamente a sua aprendizagem e percepção do cuidado.

Os estudantes relataram que a maneira como os enfermeiros e técnicos de enfermagem organizavam seu tempo e priorizavam as tarefas comunicava profissionalismo ou desorganização, influenciando a percepção de segurança no ambiente de ensino. Os profissionais que demonstravam um claro controle do tempo, realizando as checagens pré-operatórias sem pressa e o gerenciamento do fluxo de materiais de forma antecipada, eram vistos como modelos de excelência, conforme ilustrado nos depoimentos a seguir:

*O circulante sabe o que vai precisar antes mesmo de o cirurgião pedir. Isso mostra experiência e que ele valoriza o tempo: nosso e do paciente. Ele conseguia me explicar as coisas durante as pausas*. (E30)

*Quando o enfermeiro ou técnico se organiza e demonstra que tem tempo para responder uma dúvida, mesmo que seja só um minuto, comunica que o aprendizado é importante*. (E19)

*A falta de tempo, a pressa na movimentação ou a impaciência do enfermeiro quando a gente fazia uma pergunta comunicava que éramos um problema, que estávamos ali para atrapalhar*. (E7)

*Essa pressa na comunicação e no tempo para falar com a gente* [o estudante] *gera ansiedade e inibe nossa busca por esclarecimentos. Isso afeta diretamente o aprendizado*. (E25)

Percebe-se, assim, que o tempo do enfermeiro, quando bem gerido e dedicado à interação com o estudante, transforma-se em um recurso pedagógico valioso, reforçando a importância do profissional como facilitador da aprendizagem. Essa gestão temporal permite ao estudante observar, compreender e refletir sobre as práticas assistenciais, promovendo uma aprendizagem mais significativa e integrada ao contexto do cuidado.

## DISCUSSÃO

À luz dos achados, as discussões foram direcionadas ao aprofundamento do referencial teórico de Edward T. Hall^(3)^, especialmente as orientações *M-time* e *P-time*. Nesse contexto, o ambiente cirúrgico, predominantemente *M-time*, caracteriza-se por prazos rígidos, linearidade e foco na cirurgia, exigindo coordenação temporal precisa, em que desvios ou interrupções são percebidos como quebras no fluxo. Entretanto, estudantes oriundos de contextos mais *P-time* perceberam essa dinâmica temporal como um elemento comunicacional sensível, capaz de influenciar tanto a aprendizagem quanto a interação com a equipe.

Essa percepção transcende a dimensão cronológica, configurando-se como uma forma de comunicação não verbal que expressa ritmos, expectativas e modos de convivência no trabalho em saúde. No contexto da tensão entre o tempo do procedimento e o ritmo da aprendizagem, inserem-se as categorias temáticas, que detalham como essa gestão temporal se manifesta na experiência dos estudantes. Além disso, a percepção de adequação temporal reforçou o sentimento de pertencimento dos estudantes. A análise dessas percepções evidencia, assim, a profunda tensão cronêmica inerente ao ambiente de ensino-aprendizagem no ambiente.

A primeira categoria temática, “Ritmo cirúrgico como reflexo da interação da equipe”, evidencia que os estudantes perceberam o ambiente cirúrgico como um espaço caracterizado por ritmo acelerado, foco contínuo e poucas pausas, o que gerou sentimentos de estranhamento, ansiedade e insegurança durante a adaptação ao cenário^(10)^. Em alguns momentos, relataram a sensação de “ritmo quebrado”, quando demandas simultâneas alteravam o fluxo esperado da equipe.

Esses relatos reforçam que o ritmo cirúrgico, além de indicar eficiência, funciona como elemento comunicador de expectativas e regulador emocional no processo formativo, alinhando-se à perspectiva temporal *M-time* e às intrusões *P-time*, que caracterizam ambientes de alta complexidade assistencial.

O contraste entre *M-time* e *P-time* representa o núcleo da percepção do estudante sobre a cronêmica. Enquanto o ambiente cirúrgico exige rapidez e coordenação, o estudante necessita de tempo relacional e reflexivo, típico da *P-time*, para compreender o contexto e consolidar a aprendizagem. Por conseguinte, o “ritmo quebrado” pode ser compreendido como uma intrusão *P-time* na *M-time*, isto é, uma interrupção causada por demandas simultâneas que se sobrepõem à cadência esperada da equipe^(3,6)^.

Pedagogicamente, o professor e o preceptor devem mediar essa tensão, traduzindo o desvio temporal da equipe não como um sinal de caos, mas como um recurso educativo a ser analisado, orientando o estudante a interpretar essas “quebras” como momentos críticos de observação e adaptação.

A categoria temática “Espera como expressão de prioridade, tensão ou desorganização” evidencia que, em uma cultura *M-time*, a espera prolongada, quando não explicada ou justificada, comunica descaso ou falta de prioridade, gerando insegurança e percepção negativa sobre a organização do serviço^(3,8)^. “Espera é primariamente percebida” pelo estudante como falha na execução eficiente da *M-time*, mas frequentemente resulta de demandas *P-time* não gerenciadas, indicando a necessidade de comunicação intencional da equipe.

Este estudo demonstra que a cronêmica não é apenas um pano de fundo logístico, mas uma dimensão não verbal autônoma e crucial que comunica prioridade, *status* e competência. As ansiedades relatadas pelos estudantes não derivam apenas de um olhar ou da distância física inadequada, cinésica e proxêmica, mas da aceleração do ritmo ou da duração não justificada da espera. Dessa forma, esta pesquisa preenche uma lacuna ao focar no poder formativo e comunicacional do tempo, frequentemente invisível nas análises de comunicação em saúde.

O uso intencional da *P-time*, ao justificar atrasos e orientar o estudante, pode transformar a espera em tempo de observação, reduzindo interpretações equivocadas e fortalecendo o vínculo pedagógico^(1)^. Esse achado reforça a importância de empregar a *P-time* de modo estratégico, para explicar a *M-time* e mitigar interpretações negativas, garantindo que a espera seja vivenciada como tempo de aprendizagem, não de exclusão^(10)^.

Para o estudante, essa estrutura temporal representa um desafio de adaptação, pois demanda o aprendizado de um ritmo de trabalho intenso, sustentado por protocolos rígidos e pela necessidade de respostas rápidas e coordenadas. Consequentemente, a observação contínua dos modelos profissionais e a vivência prática favorecem o desenvolvimento da capacidade de interpretar sinais cronêmicos e ajustar o próprio ritmo, aprimorando competências comunicacionais^(11)^.

Influenciados pela dinâmica temporal do ambiente cirúrgico, marcada por ritmos, pausas e esperas, os participantes desenvolveram competências comunicacionais, relacionais e de autogestão do tempo, evidenciando adaptação progressiva ao contexto *M-time*, no qual o tempo se converte em linguagem e instrumento formativo.

Nesse contexto, o estudante é confrontado com uma cultura temporal diferente de outros cenários de ensino, sendo levado a desenvolver habilidades de atenção, agilidade e integração à equipe multiprofissional, sem perder de vista o sentido humano do cuidado^(12)^. Além disso, o processo de ensino-aprendizagem em ambiente cirúrgico pode ser associado à *P-time*, caracterizado pela vivência simultânea e flexível do tempo, pelas relações interpessoais, pela empatia e pela capacidade de adaptação ao contexto^(1)^.

Essa dimensão se manifesta quando o estudante começa a perceber que, para além do cumprimento das tarefas, é necessário reconhecer os ritmos humanos, o tempo da equipe em sua dinâmica de cooperação e o seu próprio tempo interno de aprendizagem e amadurecimento profissional^(10)^.

Nesse sentido, a categoria temática “Gestão do tempo pela equipe de enfermagem como modelo de cuidado e influência no aprendizado” reforça a evidência do conflito entre *M-time* e *P-time*, que reflete diretamente a tensão entre produtividade e disponibilidade relacional. A forma como o profissional aloca tempo ao ensino comunica valores, respeito, compromisso e inclusão^(3,12)^. Por outro lado, a indisponibilidade e a pressa transmitem, de modo não verbal, a exclusão do estudante da dinâmica assistencial, comprometendo sua aprendizagem^(13)^.

Os achados reforçam o papel do enfermeiro como mediador da cronêmica, responsável por equilibrar a eficiência da *M-time* com a disponibilidade relacional da *P-time*, reconhecendo o tempo como recurso pedagógico e comunicacional. Essa postura comunica uma mensagem poderosa: a produtividade não deve anular a necessidade de ensino e cuidado humanizado, transformando a gestão do tempo em um modelo de liderança inclusivo e formativo.

Além disso, o estudante é desafiado a integrar o ritmo institucional ao tempo relacional, marcado por pausas, escuta e sensibilidade. Em síntese, aprender em ambiente cirúrgico implica não apenas dominar as técnicas e fluxos, mas também articular diferentes percepções temporais, promovendo uma aprendizagem prática mais segura e consciente^(11)^.

O enfermeiro, inserido em uma lógica de precisão, deve simultaneamente adotar uma postura relacional que evidencie o desafio da gestão do tempo na prática pedagógica, onde o ritmo deixa de ser apenas um recurso e passa a funcionar como uma linguagem^(3)^. Estudos nacionais e internacionais apontam a relevância da cronêmica na comunicação profissional, embora seja menos explorada que outras dimensões não verbais, como a cinésica ou proxêmica^(14-16)^.

A percepção dos estudantes sobre a influência da cronêmica em sua ansiedade e aprendizagem reforça a necessidade de incorporar explicitamente este tema nos currículos de enfermagem, utilizando metodologias ativas. O conflito entre *M-time* e *P-time* contribui diretamente para essa prática pedagógica, ao fornecer subsídios para a formação reflexiva. Além disso, prepara o estudante para atuar eticamente em contextos de alta pressão e ritmo acelerado^(6)^. Estratégias como simulação clínica de alta fidelidade, sessões estruturadas de *debriefing* e análise reflexiva favorecem o desenvolvimento de competências comunicacionais conscientes^(6,17)^.

As dimensões do ritmo, da pausa e da espera se configuram como recursos pedagógicos e assistenciais significativos, que favorecem a identificação de necessidades de estudantes e pacientes, impactando positivamente a qualidade da assistência e a formação profissional^(8)^. Sensibilizar as equipes sobre o impacto da gestão do tempo na comunicação e adotar práticas simples, como justificar atrasos ou disponibilizar momentos de orientação, contribuem para reduzir indutores de ansiedade e fortalecer vínculos^(5,18)^.

A cronêmica, quando compreendida em profundidade, contribui para a formação de enfermeiros sensíveis, empáticos, críticos e atentos às dimensões subjetivas do cuidado^(4,18)^. A vivência temporal no ambiente cirúrgico influencia a confiança, a tomada de decisão e a identidade profissional do estudante, reforçando a importância de ambientes formativos que promovam a reflexão sobre o gerenciamento do tempo^(4,19)^.

Em contextos de alta pressão, o manejo adequado do tempo favorece a humanização do cuidado e o fortalecimento das relações interpessoais, ampliando o diálogo, a empatia, a segurança do paciente e a satisfação do estudante^(2,4)^. A escuta ativa, como experiência temporal, pode revelar lacunas organizacionais do ambiente cirúrgico que, se corrigidas, potencializam o ensino e a integração multiprofissional^(6,20)^.

Nesse contexto, a comunicação temporal não verbal deve constituir um eixo estratégico de atenção na formação de enfermeiros líderes, capazes de planejar o cuidado e organizar o tempo da equipe^(8,21)^. Ao promoverem uma gestão temporal equilibrada, tais líderes contribuem para a redução do estresse, aumentam a clareza de papéis, a qualidade e a segurança assistencial, além de fortalecerem o desenvolvimento pedagógico^(7)^.

A incorporação da cronêmica nos treinamentos de habilidades interpessoais e comunicacionais pode ampliar a consciência temporal, favorecer a coordenação de ações e respostas a situações críticas, além de contribuir para a redução de erros e acidentes^(6,21)^. Sensibilizar futuros enfermeiros para a cronêmica aprofunda a compreensão do cuidado e fortalece a dimensão humana da formação, preparando profissionais capazes de integrar eficiência técnica, sensibilidade e competência comunicacional em contextos complexos^(8)^.

### Limitações do estudo

Como limitações deste estudo, destaca-se o escopo empregado na coleta de dados, que se limitou a estudantes de enfermagem de uma única instituição pública do Norte do Brasil, o que limita a generalização dos achados. Adicionalmente, o fato de a coleta ter ocorrido em sala de aula e contar com memórias retrospectivas dos participantes resultou em um hiato temporal, mesmo que curto,entre o término das práticas no ambiente cirúrgico e as entrevistas. Isso pode ter influenciado a acurácia das lembranças e interpretações sobre a cronêmica, especialmente por se tratar de estudo voltado às percepções de tempo e ritmo.

Outra limitação reside na potencial influência do pesquisador, que é docente da instituição. Apesar das estratégias de mitigação adotadas, reconhece-se que a relação hierárquica professor-aluno pode ter gerado respostas socialmente desejáveis, configurando um viés de desejabilidade social e credibilidade. Além disso, mesmo com o hiato relativamente curto, é possível que as memórias dos estudantes tenham sido influenciadas pelo intervalo entre a prática e a entrevista, constituindo uma limitação do estudo. Por outro lado, a descrição densa do método e do contexto contribuiu para a transparência do percurso metodológico, permitindo avaliar a transferibilidade dos achados para realidades semelhantes.

### Contribuições para as áreas da enfermagem, saúde ou políticas públicas

O estudo incorpora compreensões essenciais ao campo do cuidado de enfermagem ao colocar no centro do debate um instrumento fundamental: a comunicação. Outro ponto relevante é dar voz ao estudante quanto a um aspecto pouco explorado da comunicação não verbal no ambiente cirúrgico: a importância da cronêmica para a sua formação e prática da enfermagem.

Os achados sugerem a necessidade de incluir essa temática de forma mais explícita nos currículos de enfermagem e de sensibilizar as equipes clínicas sobre o impacto comunicacional da autogestão do tempo e dos ritmos no ambiente cirúrgico. A clareza no significado das pausas, movimentos e tempos de execução pode favorecer um ambiente de ensino e trabalho mais seguro, organizado e acolhedor para os futuros enfermeiros.

Ainda assim, os resultados oferecem uma contribuição significativa ao evidenciarem a dimensão temporal da comunicação não verbal na formação em enfermagem, bem como ao discutirem suas implicações pedagógicas e assistenciais e ao ampliarem a compreensão sobre como o manejo do tempo influencia o processo de ensino-aprendizagem e a qualidade do cuidado.

## CONSIDERAÇÕES FINAIS

A compreensão do tempo, em suas diversas manifestações de ritmo, espera, duração e gestão, apresenta-se como um elemento comunicacional significativo para o cuidado de enfermagem. Os participantes perceberam a cronêmica como um indicador da dinâmica da equipe cirúrgica, da (des)organização do serviço, das prioridades do cuidado e da disponibilidade para a interação e ensino-aprendizagem, o que pode influenciar a produção de algum grau de ansiedade na experiência formativa no ambiente cirúrgico.

Diante disso, recomenda-se desenvolver simulações clínicas de alta fidelidade com foco no gerenciamento do tempo e *debriefing* estruturado para reflexão sobre o ritmo e as pausas. Além disso, sugere-se criar protocolos de comunicação temporal para equipes cirúrgicas, incentivando a justificativa de atrasos e a disponibilização de “minutos de pausa” intencionais para orientar o estudante, aprimorar suas habilidades e competências comunicacionais e aprimorar a fluidez do cuidado perioperatório.

O reconhecimento da cronêmica como recurso pedagógico essencial fortalece a formação humanística e comunicacional do futuro enfermeiro, capacitando-o para equilibrar a eficiência técnica com a sensibilidade e o cuidado centrado no outro, mesmo em ambientes de alta complexidade.

## Data Availability

Os dados de pesquisa só estão disponíveis mediante solicitação.
